# Supplementary material for: Identification, proteolytic activity quantification and biofilm-forming characterization of Gram-positive, proteolytic, psychrotrophic bacteria isolated from cold raw milk
Source: PLoS One. 2023 Sep 13;18(9):e0290953. doi: 10.1371/journal.pone.0290953 (PMC10499245; doi:10.1371/journal.pone.0290953)
Supplement: S1 Table — (DOCX) [file pone.0290953.s001.docx]

**Supplementary Information**

| **Milk sample** | **G+ count**  **CFU/ml** | **G- count**  **CFU/ml** | **Sum G+ and G-**  **CFU/ml** | **G+% Total** | **G-% Total** | **G+/G- percent** |
| --- | --- | --- | --- | --- | --- | --- |
| 1 | 68000 | 550000 | 618000 | 11.00 | 89.00 | 12.36 |
| 2 | 48000 | 340000 | 388000 | 12.37 | 87.63 | 14.12 |
| 3 | 25000 | 280000 | 305000 | 8.20 | 91.80 | 8.93 |
| 4 | 46000 | 420000 | 466000 | 9.87 | 90.13 | 10.95 |
| 5 | 31000 | 360000 | 391000 | 7.93 | 92.07 | 8.61 |
| 6 | 22000 | 190000 | 212000 | 10.38 | 89.62 | 11.58 |
| 7 | 39000 | 310000 | 349000 | 11.17 | 88.83 | 12.58 |
| 8 | 79000 | 630000 | 709000 | 11.14 | 88.86 | 12.54 |
| 9 | 11000 | 95000 | 106000 | 10.38 | 89.62 | 11.58 |
| 10 | 19000 | 140000 | 159000 | 11.95 | 88.05 | 13.57 |
| 11 | 5400 | 48000 | 53400 | 10.11 | 89.89 | 11.25 |
| 12 | 7700 | 72000 | 79700 | 9.66 | 90.34 | 10.69 |
| 13 | 86000 | 770000 | 856000 | 10.05 | 89.95 | 11.17 |
| 14 | 4300 | 59000 | 63300 | 6.79 | 93.21 | 7.29 |
| 15 | 75000 | 560000 | 635000 | 11.81 | 88.19 | 13.39 |
| 16 | 41000 | 330000 | 371000 | 11.05 | 88.95 | 12.42 |
| 17 | 51000 | 590000 | 641000 | 7.96 | 92.04 | 8.64 |
| 18 | 58000 | 430000 | 488000 | 11.89 | 88.11 | 13.49 |
| 19 | 5000 | 43000 | 48000 | 10.42 | 89.58 | 11.63 |
| 20 | 29000 | 270000 | 299000 | 9.70 | 90.30 | 10.74 |
| 21 | 37000 | 320000 | 357000 | 10.36 | 89.64 | 11.56 |
| 22 | 73000 | 550000 | 623000 | 11.72 | 88.28 | 13.27 |
| 23 | 16000 | 110000 | 126000 | 12.70 | 87.30 | 14.55 |
| 24 | 59000 | 490000 | 549000 | 10.75 | 89.25 | 12.04 |
| 25 | 14000 | 120000 | 134000 | 10.45 | 89.55 | 11.67 |
| 26 | 21000 | 380000 | 401000 | 5.24 | 94.76 | 5.53 |
| 27 | 23000 | 260000 | 283000 | 8.13 | 91.87 | 8.85 |
| 28 | 52000 | 310000 | 362000 | 14.36 | 85.64 | 16.77 |
| 29 | 78000 | 680000 | 758000 | 10.29 | 89.71 | 11.47 |
| 30 | 59000 | 460000 | 519000 | 11.37 | 88.63 | 12.83 |
| 31 | 11000 | 91000 | 102000 | 10.78 | 89.22 | 12.09 |
| 32 | 47000 | 330000 | 377000 | 12.47 | 87.53 | 14.24 |
| 33 | 33000 | 350000 | 383000 | 8.62 | 91.38 | 9.43 |
| 34 | 7100 | 79000 | 86100 | 8.25 | 91.75 | 8.99 |
| 35 | 9800 | 86000 | 95800 | 10.23 | 89.77 | 11.40 |
| 36 | 24000 | 270000 | 294000 | 8.16 | 91.84 | 8.89 |
| 37 | 35000 | 540000 | 575000 | 6.09 | 93.91 | 6.48 |
| 38 | 49000 | 380000 | 429000 | 11.42 | 88.58 | 12.89 |
| 39 | 8900 | 91000 | 99900 | 8.91 | 91.09 | 9.78 |
| 40 | 41000 | 450000 | 491000 | 8.35 | 91.65 | 9.11 |
| 41 | 48000 | 430000 | 478000 | 10.04 | 89.96 | 11.16 |
| 42 | 67000 | 560000 | 627000 | 10.69 | 89.31 | 11.96 |
| 43 | 9800 | 84000 | 93800 | 10.45 | 89.55 | 11.67 |
| 44 | 47000 | 550000 | 597000 | 7.87 | 92.13 | 8.55 |
| 45 | 11000 | 86000 | 97000 | 11.34 | 88.66 | 12.79 |
| 46 | 48000 | 540000 | 588000 | 8.16 | 91.84 | 8.89 |
| 47 | 58000 | 680000 | 738000 | 7.86 | 92.14 | 8.53 |
| 48 | 28000 | 320000 | 348000 | 8.05 | 91.95 | 8.75 |
| 49 | 53000 | 440000 | 493000 | 10.75 | 89.25 | 12.05 |
| 50 | 52000 | 480000 | 532000 | 9.77 | 90.23 | 10.83 |
| 51 | 9000 | 69000 | 78000 | 11.54 | 88.46 | 13.04 |
| 52 | 9200 | 76000 | 85200 | 10.80 | 89.20 | 12.11 |
| 53 | 23000 | 210000 | 233000 | 9.87 | 90.13 | 10.95 |
| 54 | 21000 | 190000 | 211000 | 9.95 | 90.05 | 11.05 |
| 55 | 41000 | 470000 | 511000 | 8.02 | 91.98 | 8.72 |
| 56 | 65000 | 740000 | 805000 | 8.07 | 91.93 | 8.78 |
| 57 | 31000 | 280000 | 311000 | 9.97 | 90.03 | 11.07 |
| 58 | 43000 | 540000 | 583000 | 7.38 | 92.62 | 7.96 |
| 59 | 6700 | 58000 | 64700 | 10.36 | 89.64 | 11.55 |
| 60 | 69000 | 830000 | 899000 | 7.68 | 92.32 | 8.31 |
| 61 | 12000 | 88000 | 100000 | 12.00 | 88.00 | 13.64 |
| 62 | 4000 | 27000 | 31000 | 12.90 | 87.10 | 14.81 |
| 63 | 39000 | 580000 | 619000 | 6.30 | 93.70 | 6.72 |
| 64 | 43000 | 450000 | 493000 | 8.72 | 91.28 | 9.56 |
| 65 | 7400 | 67000 | 74400 | 9.95 | 90.05 | 11.04 |
| 66 | 4300 | 22000 | 26300 | 16.35 | 83.65 | 19.55 |
| 67 | 16000 | 92000 | 108000 | 14.81 | 85.19 | 17.39 |
| 68 | 20000 | 170000 | 190000 | 10.53 | 89.47 | 11.76 |
| 69 | 8000 | 69000 | 77000 | 10.39 | 89.61 | 11.59 |
| 70 | 26000 | 180000 | 206000 | 12.62 | 87.38 | 14.44 |
| 71 | 21000 | 260000 | 281000 | 7.47 | 92.53 | 8.08 |
| 72 | 39000 | 480000 | 519000 | 7.51 | 92.49 | 8.13 |
| 73 | 9500 | 73000 | 82500 | 11.52 | 88.48 | 13.01 |
| 74 | 18000 | 160000 | 178000 | 10.11 | 89.89 | 11.25 |
| 75 | 8900 | 83000 | 91900 | 9.68 | 90.32 | 10.72 |
| 76 | 41000 | 290000 | 331000 | 12.39 | 87.61 | 14.14 |
| 77 | 59000 | 530000 | 589000 | 10.02 | 89.98 | 11.13 |
| 78 | 33000 | 390000 | 423000 | 7.80 | 92.20 | 8.46 |
| 79 | 49000 | 410000 | 459000 | 10.68 | 89.32 | 11.95 |
| 80 | 9400 | 55000 | 64400 | 14.60 | 85.40 | 17.09 |
| 81 | 31000 | 170000 | 201000 | 15.42 | 84.58 | 18.24 |
| 82 | 8400 | 41000 | 49400 | 17.00 | 83.00 | 20.49 |
| 83 | 13000 | 150000 | 163000 | 7.98 | 92.02 | 8.67 |
| 84 | 83000 | 360000 | 443000 | 18.74 | 81.26 | 23.06 |
| 85 | 30000 | 180000 | 210000 | 14.29 | 85.71 | 16.67 |
| 86 | 37000 | 250000 | 287000 | 12.89 | 87.11 | 14.80 |
| 87 | 9100 | 78000 | 87100 | 10.45 | 89.55 | 11.67 |
| 88 | 43000 | 190000 | 233000 | 18.45 | 81.55 | 22.63 |
| 89 | 3700 | 29000 | 32700 | 11.31 | 88.69 | 12.76 |
| 90 | 6900 | 88000 | 94900 | 7.27 | 92.73 | 7.84 |
| 91 | 35000 | 240000 | 275000 | 12.73 | 87.27 | 14.58 |
| 92 | 3000 | 28000 | 31000 | 9.68 | 90.32 | 10.71 |
| 93 | 47000 | 540000 | 587000 | 8.01 | 91.99 | 8.70 |
| 94 | 49000 | 420000 | 469000 | 10.45 | 89.55 | 11.67 |
| 95 | 27000 | 190000 | 217000 | 12.44 | 87.56 | 14.21 |
| 96 | 81000 | 590000 | 671000 | 12.07 | 87.93 | 13.73 |
| 97 | 84000 | 680000 | 764000 | 10.99 | 89.01 | 12.35 |
| 98 | 7200 | 48000 | 55200 | 13.04 | 86.96 | 15.00 |
| 99 | 19000 | 120000 | 139000 | 13.67 | 86.33 | 15.83 |
| 100 | 67000 | 780000 | 847000 | 7.91 | 92.09 | 8.59 |
| **MEAN** | **33397** | **301150** | **334547** | **9.98** | **90.02** | **11.09** |
