# Supplementary material for: Identification, proteolytic activity quantification and biofilm-forming characterization of Gram-positive, proteolytic, psychrotrophic bacteria isolated from cold raw milk
Source: PLoS One. 2023 Sep 13;18(9):e0290953. doi: 10.1371/journal.pone.0290953 (PMC10499245; doi:10.1371/journal.pone.0290953)
Supplement: Attachment — Submitted filename: PONE-D-23-16130.pdf [file pone.0290953.s002.pdf]

Reviewer Comments

Manuscript PONE-D-23-16130

In this study, the authors aimed to isolate and characterize Gram-positive, proteolytic and psychrotrophic bacteria from raw milk. The enzymatic activities and formation of biofilms are very exploited abilities in Gram-negative contaminants of milk, which can be considered a differential of the present study. The contextualization of the problem in the Introduction does not bring anything new and is weakly supported by outdated references. Only 17% (4) of the references are from the last five years, and the most recent are from 2020. It would be interesting if the authors addressed in the Introduction the reason that led them to analyze Gram-positive bacteria, which according to the authors, constitute approximately 10% of the microbial population in cold raw milk that has been stored. The experimental strategy is simple, adopts basic and conventional techniques for isolating and forming biofilms, and uses molecular techniques (16S rRNA) for isolate identification.

General Comments

- An update of bibliographic references is strongly needed. The subject is widely studied, and there are many recent publications.
- Standardization is required. For example, there are two forms of volume units: ml and mL.

Specific Comments

Line 62: sensorial properties instead of organoleptic properties

Lines 71-73: Why are these few studies on Gram-positive psychrotrophic bacteria not mentioned here? This is the subject of this study and was not explored in the Introduction. What are the genera of proteolytic
psychrotrophic bacteria found in milk? Do they form biofilms?
Information about it is not considered in the context of the addressed problem.

Line 91: Was the sequence not analyzed from 16S rDNA? How was the DNA extracted? Inform about the methods, reagents, or kits used and the referred commercial brands.

Line 101: The centrifugation detail should be informed in centrifuge force (g), not rpm.

Lines 138-150: All this information belongs to the Introduction section. There are no results or discussion here.

Line 140: Please check the accuracy of this information in the cited references.

Line 190: A thorough literature review should be done before making this statement

Line 198: In many situations, the biofilm production by certain bacteria is only verified when the substrate is periodically changed.

Line 210: Although there is no consensus on the number of cells present on the surface to be considered a biofilm, this value of 1.34 log cfu/cm2 is too low and is not considered a biofilm.

Line 237: A careful standardization of the reference section is necessary.
