# Supplementary material for: Identification, proteolytic activity quantification and biofilm-forming characterization of Gram-positive, proteolytic, psychrotrophic bacteria isolated from cold raw milk
Source: PLoS One. 2023 Sep 13;18(9):e0290953. doi: 10.1371/journal.pone.0290953 (PMC10499245; doi:10.1371/journal.pone.0290953)
Supplement: Attachment — Submitted filename: Responses to the Reviewers Comments.docx [file pone.0290953.s003.docx]

**Responses to the Respected Editor and Reviewers' Comments:**

**Editor's comment:**

**Comment 1:** Please ensure that your manuscript meets PLOS ONE's style requirements, including those for file naming. The PLOS ONE style templates can be found at

https://journals.plos.org/plosone/s/file?id=wjVg/PLOSOne_formatting_sample_main_body.pdf and

https://journals.plos.org/plosone/s/file?id=ba62/PLOSOne_formatting_sample_title_authors_affiliations.pdf

**Response 1:** Thank you so much. It was checked again and carefully.

**Comment 2:** We noticed you have some minor occurrence of overlapping text with the following previous publication(s), which needs to be addressed:

https://linkinghub.elsevier.com/retrieve/pii/S0958694620301576

https://dokumen.pub/microbiology-in-dairy-processing-challenges-and-opportunities-9781119114802-1119114802-9781119114970-1119114977-9781119114987-1119114985.html

In your revision ensure you cite all your sources (including your own works), and quote or rephrase any duplicated text outside the methods section. Further consideration is dependent on these concerns being addressed.

**Response 1:** Thank you so much. You have mentioned an article and a book. The mentioned article, which is one of my own articles, is in the reference list of this manuscript. Before submitting this manuscript, I did my best to include that text in a rephrased form. If there are still some sentences that need to be rephrased, I'd appreciate it if you could let me know those sentences.

However, in the case of the mentioned book, that book was not used in writing this manuscript. Maybe there was a sentence from somewhere else or in my mind as the author, the main source of which is this book, but I did not know about it. If there are some sentences that need to be rephrased, I'd appreciate it if you could let me know those sentences.

**Comment 3:** We suggest you thoroughly copyedit your manuscript for language usage, spelling, and grammar. If you do not know anyone who can help you do this, you may wish to consider employing a professional scientific editing service.

Upon resubmission, please provide the following:

The name of the colleague or the details of the professional service that edited your manuscript

A copy of your manuscript showing your changes by either highlighting them or using track changes (uploaded as a *supporting information* file)

A clean copy of the edited manuscript (uploaded as the new *manuscript* file)

**Response 3:** Thank you so much. Before submitting this manuscript to PLOS One, we submitted that to Language revision services at the University of Helsinki. (<https://www.helsinki.fi/en/language-centre/cooperation-and-language-services/language-revision-services-at-the-university-of-helsinki>).

They edited the manuscript for English writing. I will submit the edited manuscript as a supporting information file.

**Comment 4:** Thank you for stating the following in the Acknowledgments Section of your manuscript:

“A research grant (SCU.VF99.245) provided by Shahid Chamran University of Ahvaz supported this study. In addition, The Finnish Food Research Foundation and The Finnish Society of Sciences and Letters are acknowledged for awarding grants to Amin Yousefvand on the dates 16.03.2021 and 28.03.2022. The open access funded by Helsinki University Library.”

We note that you have provided funding information that is not currently declared in your Funding Statement. However, funding information should not appear in the Acknowledgments section or other areas of your manuscript. We will only publish funding information present in the Funding Statement section of the online submission form.

Please remove any funding-related text from the manuscript and let us know how you would like to update your Funding Statement. Currently, your Funding Statement reads as follows:

“The author(s) received no specific funding for this work.”

Please include your amended statements within your cover letter; we will change the online submission form on your behalf.

**Response 4:** Funding information was removed from the acknowledgment and added to the cover letter.

**Comment 5:** In your Data Availability statement, you have not specified where the minimal data set underlying the results described in your manuscript can be found. PLOS defines a study's minimal data set as the underlying data used to reach the conclusions drawn in the manuscript and any additional data required to replicate the reported study findings in their entirety. All PLOS journals require that the minimal data set be made fully available. For more information about our data policy, please see http://journals.plos.org/plosone/s/data-availability.

"Upon re-submitting your revised manuscript, please upload your study’s minimal underlying data set as either Supporting Information files or to a stable, public repository and include the relevant URLs, DOIs, or accession numbers within your revised cover letter. For a list of acceptable repositories, please see http://journals.plos.org/plosone/s/data-availability#loc-recommended-repositories. Any potentially identifying patient information must be fully anonymized.

Important: If there are ethical or legal restrictions to sharing your data publicly, please explain these restrictions in detail. Please see our guidelines for more information on what we consider unacceptable restrictions to publicly sharing data: http://journals.plos.org/plosone/s/data-availability#loc-unacceptable-data-access-restrictions. Note that it is not acceptable for the authors to be the sole named individuals responsible for ensuring data access.

We will update your Data Availability statement to reflect the information you provide in your cover letter.

**Response 5:** Supplementary information files were prepared and submitted.

**Comment 6:** Please include a separate caption for each figure in your manuscript.

**Response 6:** A separate caption for the figure was included in the manuscript.

**Reviewers' comments:**
**Reviewer #1**

**Comment 1**: The topic of the manuscript (Gram-positive psychrotrophic bacteria in raw milk, their proteolytic activity and biofilm-forming abilities) has not been well recognized until now, therefore the results presented in the manuscript would be of interest to the readers. The manuscript presents in a clear manner the subject, the methodological part is described clearly and mostly detailed enough.

**Response 1:** Thank you so much.

**Comment 2:** The biggest disadvantage of the manuscript is the lack of any characteristics of the milk samples taken for analysis. I understand that the main goal of the work was focused on the isolation and characteristics of the G+PP bacteria, however, at least basic information on the raw material should be added e.g. total bacterial number, numbers of mesophilic and psychroptophic bacteria, proportions of Gram-positive to Gram-negative bacteria in the population of the psychrotrophic ones. This information (particularly the latter one) would enable the Authors to elaborate on the importance of their work for the dairy industry.

**Response 2:** Thanks for your good suggestions. Unfortunately, we do not have information about the total number and the number of mesophilic bacteria in the milk samples because the collection of this information was not in line with the goals of our research. As you know, the focus of this study was on psychrotrophic bacteria, so the incubations were done only for this category of bacteria. We have the information on the proportions of Gram-positive to Gram-negative bacteria in the population of the psychrotrophic bacteria. However, considering that this article specifically deals with the subject of psychrotrophic, proteolytic bacteria and the ratio of psychrotrophic proteolytic gram-positive bacteria to psychrotrophic proteolytic gram-negative bacteria has presented in the results section, in order to avoid confusion of the readers and interference of topics, the desired information of the respected reviewer is presented as supplementary information. I hope it will be accepted by the respected reviewer.

**Comment 3:** 16S rRNA gene sequences should be deposited in a public repository, e.g. in GenBank and accession numbers should be placed in the manuscript

**Response 3:** Thanks for your good suggestions. Based on this comment, table 1 was revised and the desired information was added

**Comment 4:** Line 83-84 Please give information on the season when the samples were taken and if it influences the bacterial composition of raw milk in Iran. You can include this information here or in the discussion section.

**Response 4:** Thank you for your good suggestions. All the samples were collected during winter. This information was added to the text.

**Comment 5:** Line 124 Please give the inoculation level

**Response 5:** Thank you so much. The inoculation level was added to the text.

**Comment 6:** Line 133-136 Please give information on statistical tests applied for data analysis, include also the requirements that data had to meet to be processed with the tests.

**Response 6:** Thank you so much. This information was added to the text.

**Comment 7:** Line156 Please give information on whether the 36 G+PP isolates were from different sampling locations/milk samples. Please discuss the prevalence of G+PP bacteria among psychrotrophic bacteria and their (G+PP bacteria) possible impact on the milk production process.

**Response 7:** Thank you so much. This information was added to the text.

**Reviewer #2**

**Comment 1:** In this study, the authors aimed to isolate and characterize Gram-positive, proteolytic and psychrotrophic bacteria from raw milk. The enzymatic activities and formation of biofilms are very exploited abilities in Gram-negative contaminants of milk, which can be considered a differential of the present study. The contextualization of the problem in the Introduction does not bring anything new and is weakly supported by outdated references. Only 17% (4) of the references are from the last five years, and the most recent are from 2020. It would be interesting if the authors addressed in the Introduction the reason that led them to analyze Gram-positive bacteria, which according to the authors, constitute approximately 10% of the microbial population in cold raw milk that has been stored.

**Response 1:** Thanks for your good suggestions. Introduction of the manuscript was revised based on this comment.

**Comment 2:** An update of bibliographic references is strongly needed. The subject is widely studied, and there are many recent publications.

**Response 2:** The entire manuscript was revised accordingly and recent publications were added to the text.

**Comment 3:** Standardization is required. For example, there are two forms of volume units: ml and mL.

**Response 3:** It was corrected in the text.

**Comment 4:** Line 62: sensorial properties instead of organoleptic properties

**Response 4:** It was corrected in the text.

**Comment 5:** Lines 71-73: Why are these few studies on Gram-positive psychrotrophic bacteria not mentioned here? This is the subject of this study and was not explored in the Introduction. What are the genera of proteolytic psychrotrophic bacteria found in milk? Do they form biofilms? Information about it is not considered in the context of the addressed problem.

**Response 5:** Thanks for your good suggestions. Introduction was revised accordingly and new information was added.

**Comment 6:** Line 91: Was the sequence not analyzed from 16S rDNA? How was the DNA extracted? Inform about the methods, reagents, or kits used and the referred commercial brands.

**Response 6:** Thank you so much. The resulting PCR products were extracted from agarose gel using Gel DNA Recovery Kit-EX6151 (SinaClon BioSciences, Iran), and sequenced with a 16S forward primer using a 310 automatic DNA Sequencer (Applied Biosystems, Foster City, CA, USA). This information was added to the text.

**Comment 7:** Line 101: The centrifugation detail should be informed in centrifuge force (g), not rpm.

**Response 7:** Thank you so much. It was corrected in the text.

**Comment 8:** Lines 138-150: All this information belongs to the Introduction section. There are no results or discussion here.

**Response 8:** Thanks for your good suggestion. The text was revised accordingly.

**Comment 9:** Line 140: Please check the accuracy of this information in the cited references.

**Response 9:** Thank you so much. This information was deleted in the revised manuscript.

**Comment 10:** Line 190: A thorough literature review should be done before making this statement

**Response 10:** Your point is absolutely right. We again searched the available sources. indeed, very few studies have been done on the biofilm formation of Gram-positive psychrotrophic bacteria.

**Comment 11:** Line 198: In many situations, the biofilm production by certain bacteria is only verified when the substrate is periodically changed.

**Response 11:** My apologies, but I don't understand what the respected reviewer means and its relevance to this research. If it is stated more clearly, it will be considered in the revised manuscript.

**Comment 12:** Line 210: Although there is no consensus on the number of cells present on the surface to be considered a biofilm, this value of 1.34 log cfu/cm2 is too low and is not considered a biofilm.

**Response 12:** Thank you so much. I understand your point, but I draw your attention to the fact that all plates were washed three times before quantifying the biofilm. Therefore, this low number of bacteria are definitely attached to the surface and so, we should consider them as biofilms.

**Comment 13:** Line 237: A careful standardization of the reference section is necessary.

**Response 13:** Thank you so much. References were checked again and carefully.
