# Supplementary material for: Identification, proteolytic activity quantification and biofilm-forming characterization of Gram-positive, proteolytic, psychrotrophic bacteria isolated from cold raw milk
Source: PLoS One. 2023 Sep 13;18(9):e0290953. doi: 10.1371/journal.pone.0290953 (PMC10499245; doi:10.1371/journal.pone.0290953)
Supplement: Attachment — Submitted filename: Responses to the Reviewers Comments-R2.docx [file pone.0290953.s004.docx]

**Responses to the Respected Reviewers' Comments:**

**Comment 1:** Ref. to Comment 4: “Please give information on the season when the samples were taken and if it influences the bacterial composition of raw milk in Iran. You can include this information here or in the discussion section.” Please comment on how the season of milk collection influences the microbiological composition of milk in Iran. If there is no data on this, please also include this information.

**Response 1:** Thank you so much. In this region, the weather is mild in autumn and winter and hot in spring and summer. Therefore, in the months with moderate temperature, the total bacterial count of raw milk is less. nevertheless, after cooling milk and during refrigerated storage, the population of psychrotrophic bacteria increases, as expected. This information was added to the text.

**Comment 2:** Line 56 “poor quality” of what?

**Response 1:** Thank you so much. It was corrected in the text: poor quality of dairy products.

**Comment 3:** Line 153 Please add information on the % similarity to the species reference sequence obtained for sequences obtained in this study. You may give the threshold of the %similarity applied for the species identification. You may give the information here or in Table 1.

**Response 3:** Thank you so much. More than 97% similarity level was considered for bacterial species identification. This information was added to the text.

**Comment 4:** Line 158 “predominant genera. Subsequently”

**Response 4:** Thank you so much. It was corrected.

**Comment 5:** Supplementary Information – please give the units of the data. Is it colony forming units per milliliter?

**Response 5:** Thank you so much. Yes, it is CFU/ml. It was corrected in the Supplementary Information.
